# The Naval Medical Examination Board

**Published:** 1853-04

**Authors:** 


The Naval Medical Examination Board met at Philadelphia on the 15th of December, and has reported the following Assistant Surgeons as qualified for promotion: 1, Wm. Lowber; 2, P. J. Horwitz; 3, Rush Mitchell; 4, D. E. Phillips; 5, Jas. Hamilton; 6, J. L. Burtt. Thirty-four candidates for admission into the Navy as Assistant-Surgeons, were examined, and the following nine were selected as the best qualified; 1, James H. Stuart, of Pennsylvania; 2, J. Pembroke Thorn, of Virginia; 3, John M. Browne, of New Hampshire; 4, John T. Taylor, of Delaware; 5. Henry Clay Caldwell, of Virginia: 6, Thomas J. Turner, of Pennsylvania; 7, William T. Hord, of Kentucky; 8, Wentworth R. Richardson, of Massachusetts; 9, A. Clarkson Smith, of Pennsylvania.

